# Data on analysis of coronary atherosclerosis on computed tomography and ^18^F-sodium fluoride positron emission tomography

**DOI:** 10.1016/j.dib.2017.06.011

**Published:** 2017-06-12

**Authors:** Toshiro Kitagawa, Hideya Yamamoto, Shinya Toshimitsu, Ko Sasaki, Atsuhiro Senoo, Yumiko Kubo, Fuminari Tatsugami, Kazuo Awai, Yutaka Hirokawa, Yasuki Kihara

**Affiliations:** a Department of Cardiovascular Medicine, Hiroshima University Graduate School of Biomedical and Health Sciences, Hiroshima, Japan; b Hiroshima Heiwa Clinic, Hiroshima, Japan; c Department of Diagnostic Radiology, Hiroshima University Hospital, Hiroshima, Japan

## Abstract

This article contains the data showing illustrative examples of plaque classification on coronary computed tomography angiography (CCTA) and measurement of ^18^F-sodium fluoride (^18^F-NaF) uptake in coronary atherosclerotic lesions on positron emission tomography (PET). We divided the lesions into one of three plaque types on CCTA (calcified plaque, non-calcified plaque, partially calcified plaque). Focal ^18^F-NaF uptake of each lesion was quantified using maximum tissue-to-background ratio. This article also provides a representative case with a non-calcified coronary plaque detected on CCTA and identified on ^18^F-NaF PET/non-contrast computed tomography based on a location of a vessel branch as a landmark. These complement the data reported by Kitagawa et al. (2017) [1].

**Specifications Table**

**Table:** 

Subject area	Medicine
More specific subject area	Cardiovascular imaging
Type of data	Clinical images (computed tomography, positron emission tomography)
How data was acquired	Computed tomography scan (Aquilion One; Toshiba Medical Systems, Tokyo, Japan), positron emission tomography scan (Discovery ST Elite-Performance; GE Healthcare, Waukesha, WI, USA)
Data format	Raw and analyzed data
Experimental factors	The data for reconstruction of clinical images were acquired with electrocardiogram-gated scan.
Experimental features	All reconstructed image data were transferred and analyzed with offline workstations.
Data source location	Hiroshima, Japan
Data accessibility	Data are with this article

**Value of the data**

• The data represent the illustrative examples of plaque classification (calcified plaque, non-calcified plaque, partially calcified plaque) on coronary computed tomography angiography (CCTA).
• The data explain how to assess ^18^F-sodium fluoride (^18^F-NaF) uptake in coronary atherosclerotic lesions on positron emission tomography (PET).
• The data provide a representative case with a non-calcified coronary plaque detected on CCTA and identified on ^18^F-NaF PET/non-contrast computed tomography based on a location of a vessel branch as a landmark.

## 1 Data

**Fig. 1:**
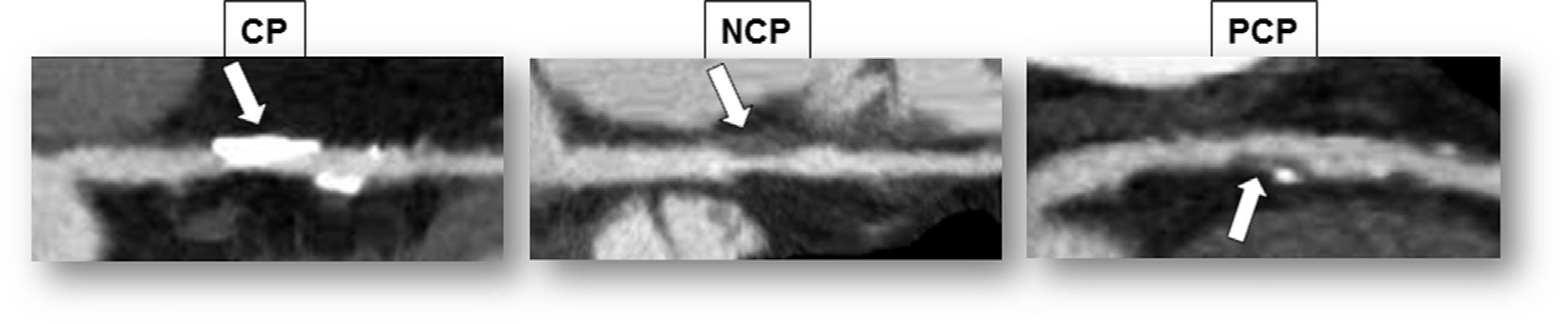
Three plaque types of coronary atherosclerotic lesions on CCTA.

**Fig. 2:**
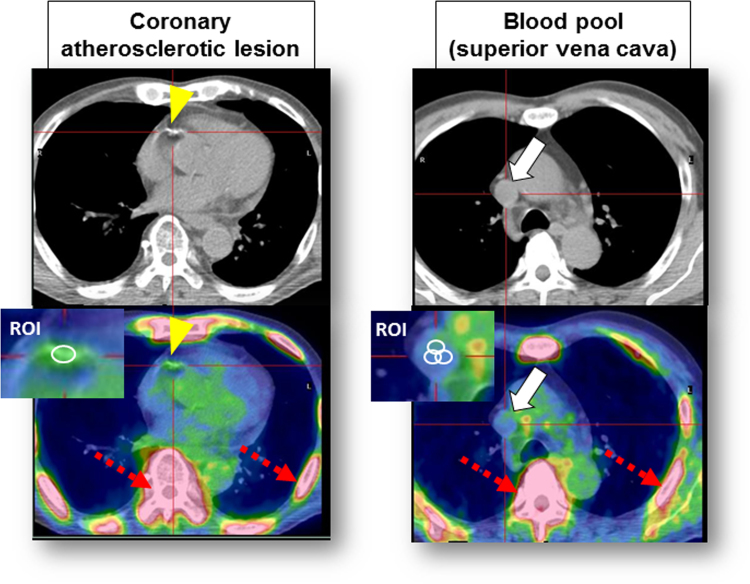
Measurements of ^18^F-NaF uptake of coronary atherosclerotic lesion (arrowheads) and blood pool (arrows) on PET/CT images. Dot-arrows indicate the intense uptake in the vertebrae and ribs.

**Fig. 3:**
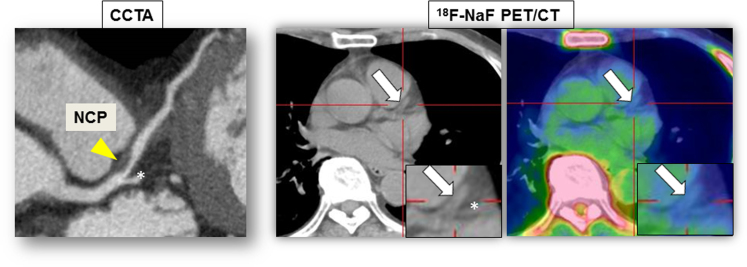
A 75-year-old male had a NCP in the proximal portion of the left anterior descending artery on CCTA (arrowhead). Its location on ^18^F-NaF PET/non-contrast CT (arrows) was determined using a vessel branch as a landmark (asterisks). The fused PET/CT image showed minimal ^18^F-NaF uptake corresponding to the NCP (TBR_max_ = 0.88).

The data presented include the illustrative examples of plaque classification on CCTA (Fig. 1) and measurement of ^18^F-NaF uptake in coronary atherosclerotic lesions on PET (Fig. 2), and images of a case with a non-calcified coronary plaque detected on CCTA and identified on ^18^F-NaF PET/non-contrast computed tomography (CT) (Fig. 3).

These complement the data reported by Kitagawa et al. (2017) [1].

## 2 Experimental design, materials and methods

### 2.1 Study participants

We prospectively recruited 32 patients with known or suspected coronary artery disease (CAD) who underwent both cardiac CT and ^18^F-NaF PET/CT between June 2014 and August 2016. To be included in the ^18^F-NaF PET/CT study, the patient had to have at least one coronary atherosclerotic lesion detected on CCTA in segments >2-mm in diameter according to the Society of Cardiovascular Computed Tomography׳s 18-segment model [2]. Our hospital׳s ethics committee approved the study protocol, and written informed consent was obtained from all patients.

### 2.2 Cardiac CT scan and image analysis

Cardiac CT scan was performed using a 320-slice CT scanner (Aquilion One; Toshiba Medical Systems, Tokyo, Japan). The data set for CCTA was acquired using the HeartNAVI® system (collimation 320×0.5 mm; tube current 350–580 mA; tube voltage 120 kV; Toshiba Medical Systems) with retrospective electrocardiography gating. All reconstructed CT image data were transferred to an offline workstation (Advantage Workstation Ver. 4.2, GE Healthcare, Waukesha, WI, USA) for post-processing and image analysis.

On CCTA, we divided coronary atherosclerotic lesions into one of three plaque types (Fig. 1): calcified plaque (CP), containing only a structure on the vessel wall with a CT density above that of the contrast-enhanced coronary lumen or with a CT density of >130 Hounsfield units (HU); non-calcified plaque (NCP), containing only a low-density mass >1 mm^2^ in size, located within the vessel wall and clearly distinguishable from the contrast-enhanced coronary lumen and the surrounding pericardial tissue; partially calcified plaque (PCP), containing both CP and NCP components.

### 2.3 18F-NaF PET/CT protocol and image analysis

Combined PET/CT imaging of coronary arteries and superior vena cava was performed using a hybrid scanner (Discovery ST Elite-Performance, GE Healthcare) within 1 month after cardiac CT imaging. The patients were administered a target dose of 370 MBq ^18^F-NaF intravenously and subsequently rested in a quiet environment for 60 min. Initially, a non-enhanced CT scan was performed for attenuation correction of the PET images, and then an electrocardiogram-gated emission PET scan of the thorax was performed with a 25-min acquisition using the three-dimensional mode. Immediately after the PET scan, an electrocardiogram-gated CT scan for fusion with PET images was performed with the same axial coverage at 120 kV, 250 mA, a speed of 27.0 mm/rotation, and a slice thickness 1.25 mm. The PET component of the combined imaging system allows simultaneous acquisition of 47 transaxial PET images with an interslice spacing of 3.27 mm in the one bed position. The PET data were reconstructed with an ordered-subset expectation maximization iterative reconstruction algorithm called as VUE Point Plus (21 subsets and two iterations).

The PET data were reconstructed in 8 multiple phases of the cardiac cycle, with the diastolic phase (62.5–75%) used for analysis with an offline workstation (Advantage Workstation Ver. 4.4, GE Healthcare). For ^18^F-NaF uptake for coronary atherosclerotic lesions, a region of interest (ROI) was drawn around each lesion on 3.27 mm axial slices just beyond the discernible adventitial border. The maximum SUV (SUV_max_) (decay-corrected tissue concentration of the tracer divided by the injected dose per body weight) was measured and divided by an averaged mean SUV in the blood pool, derived from five circular ROIs positioned in the center of the superior vena cava (Fig. 2). This provided a maximum tissue-to-background ratio (TBR_max_), which was reported as a measure of ^18^F-NaF uptake for each coronary atherosclerotic lesion.

### 2.4 Representative case with a non-calcified coronary plaque

Unlike CP and PCP, NCP detected on CCTA cannot be identified by locations of vessel calcium on non-contrast CT image. Thus, the identification of NCP on ^18^F-NaF PET/non-contrast CT was based on locations of vessel branches as landmarks. Fig. 3 shows a case with NCP detected on CCTA and analyzed on ^18^F-NaF PET/non-contrast CT.

## Conflict of interest

The authors declared they do not have anything to disclose regarding conflict of interest with respect to this manuscript.
